# A Germline‐Specific Regulator of Mitochondrial Fusion is Required for Maintenance and Differentiation of Germline Stem and Progenitor Cells

**DOI:** 10.1002/advs.202203631

**Published:** 2022-10-18

**Authors:** Ru Zhang, Yi‐Xuan Tu, Ding Ye, Zhenglong Gu, Zhen‐Xia Chen, Yonghua Sun

**Affiliations:** ^1^ State Key Laboratory of Freshwater Ecology and Biotechnology Institute of Hydrobiology Innovation Academy for Seed Design Chinese Academy of Sciences Wuhan 430072 China; ^2^ Hubei Key Laboratory of Agricultural Bioinformatics College of Life Science and Technology College of Biomedicine and Health Interdisciplinary Sciences Institute Huazhong Agricultural University Wuhan 430070 China; ^3^ Hubei Hongshan Laboratory Wuhan 430070 China; ^4^ Division of Nutritional Sciences Cornell University Ithaca NY 14853 USA; ^5^ Center for Mitochondrial Genetics and Health Greater Bay Area Institute of Precision Medicine (Guangzhou) Fudan University Nansha District Guangzhou 511400 China; ^6^ Shenzhen Institute of Nutrition and Health Huazhong Agricultural University Shenzhen 518000 China; ^7^ Shenzhen Branch Guangdong Laboratory for Lingnan Modern Agriculture Genome Analysis Laboratory of the Ministry of Agriculture Agricultural Genomics Institute at Shenzhen Chinese Academy of Agricultural Sciences Shenzhen 518000 China

**Keywords:** gametogenesis, germline stem cells, mitochondrial dynamics, *Pld6*, progenitor cells

## Abstract

Maintenance and differentiation of germline stem and progenitor cells (GSPCs) is important for sexual reproduction. Here, the authors identify zebrafish *pld6* as a novel germline‐specific gene by cross‐analyzing different RNA sequencing results, and find that *pld6* knockout mutants develop exclusively into infertile males. In *pld6* mutants, GSPCs fail to differentiate and undergo apoptosis, leading to masculinization and infertility. Mitochondrial fusion in *pld6*‐depleted GSPCs is severely impaired, and the mutants exhibit defects in piRNA biogenesis and transposon suppression. Overall, this work uncovers zebrafish *Pld6* as a novel germline‐specific regulator of mitochondrial fusion, and highlights its essential role in the maintenance and differentiation of GSPCs as well as gonadal development and gametogenesis.

## Graphical abstract

Germline stem and progenitor cells (GSPCs) are important for sexual reproduction. A novel germline‐specific gene, *pld6*, encoding a mitochondrial fusion regulator in zebrafish, is identified and studied by CRISPR/cas9‐mediated gene knockout. This is the first discovery of a germline‐specific mitochondrial fusion regulator that is essential for the maintenance and differentiation of GSPCs and thus for gonadal development and gametogenesis.

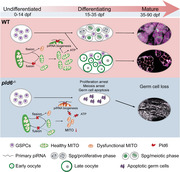

## 1 Introduction

The mechanisms of sex determination are amazingly diverse. One mechanism is genetic sex determination, such as the XX/XY sex chromosome system in mammals and the ZZ/ZW sex chromosome system in birds.^[^ ^1^, ^2^ ^]^ Another mechanism is environmental sex determination (ESD), such as temperature controlled sex determination.^[^ ^3^ ^]^ In laboratory strains of zebrafish, sex differentiation is controlled by both polygenes and environmental factors, with the heteromorphic sex chromosome lost during domestication in the laboratory.^[^ ^4^ ^]^ Although adult zebrafish have two differentiated sexes, they all initially develop as ovary‐like juvenile gonads called “bipotential juvenile ovaries”. Zebrafish juvenile gonads contain immature oocytes capable of producing two end‐differentiated cell types, sperm, or oocytes. During development, approximately half of the juveniles undergo oogenesis and become females, while the other half terminates oogenesis and finally develop into males. The juvenile ovary‐to‐testis transformation in putative males arises from apoptosis‐driven degeneration of immature oocytes at about 23–35 days post fertilization (dpf).^[^ ^5^, ^6^ ^]^ Germline stem and progenitor cells (GSPCs), including primordial germ cells (PGCs) and germline stem cells (GSCs), greatly affects the sexual fate decision of germ cells. For example, an abundance of PGCs promotes ovary development, and insufficiency of PGCs promotes testis formation.^[^ ^7^, ^8^ ^]^ GSCs continue to proliferate throughout reproductive life of organisms to generate cells that undergo differentiation, while self‐renewing themselves.^[^ ^9^, ^10^ ^]^

The formation, maintenance, and differentiation of GSPCs are regulated by a multitude of mechanisms. In zebrafish, germ cells are set aside early on during embryogenesis as PGCs, by maternally‐derived germ plasm components.^[^ ^11^ ^]^ Disruption of germ plasm components, such as dead end (Dnd) and tudor domain‐containing proteins (TDRDs) usually resulted in the decrease of PGCs leading to masculinization and infertility.^[^ ^12^, ^13^, ^14^, ^15^, ^16^ ^]^ The PGCs then migrate to the developing gonad by following a gradient of the SDF1‐CXCR signaling.^[^ ^17^ ^]^ Disruption of factors involved in PGC migration, such as *sdf1a*,^[^ ^18^ ^]^ *rgs14a*,^[^ ^19^ ^]^ and *ca15b*,^[^ ^20^, ^21^ ^]^ also lead to sterilization.^[^ ^22^, ^23^ ^]^ The GSPC proliferation and differentiation are modulated by changes at the level of translation, transcription, RNA processing, as well as epigenetic modifications. The conserved NANOS translational repressors are essential to prevent premature germ cell differentiation.^[^ ^24^, ^25^, ^26^ ^]^ Vasa, which is a RNA‐helicase and promotes translation, is also required for GSPC differentiation and maintenance.^[^ ^27^ ^]^ PIWI‐interacting RNAs (piRNAs) contribute to both GSPC maintenance and to orchestrate gametogenesis by ensuring the stability of germline genome.^[^ ^28^, ^29^ ^]^ In mice, mutation of DNA methyltransferase (DNMT) induced accumulation of GSCs in males, namely spermatogonial stem cells (SSCs).^[^ ^30^ ^]^ Moreover, germline‐specific expression pattern is likely to be observed in these factors which are essential for GSPC specification, migration, self‐renewal, as well as differentiation.^[^ ^31^, ^32^, ^33^ ^]^

In addition to their long‐recognized role in energy production, mitochondria may also regulate cell fate determination and differentiation.^[^ ^34^ ^]^ The balance of mitochondrial fusion and fission, namely mitochondrial dynamics, is tightly related to cellular physiology and even cell fate.^[^ ^35^, ^36^ ^]^ It has been reported that normal mitochondrial dynamics is crucial for self‐renewal and differentiation of GSCs in *Drosophila*. For example, disrupted mitochondrial fusion impacts GSC maintenance by interfering lipid metabolism in a cell‐autonomous manner,^[^ ^37^ ^]^ and mitochondrial fission regulates germ cell differentiation by suppressing reactive oxygen species‐mediated activation of epidermal growth factor signaling.^[^ ^38^ ^]^ Mitochondria undergo continuous morphological and distributional changes during spermatogenesis^[^ ^39^ ^]^ and metabolic changes induced by mitochondrial function accompany SSC differentiation.^[^ ^40^ ^]^ However, it remains unknown whether there exists a germline‐specific regulator of mitochondrial dynamics, and how it functions in germ cell development and gonadal differentiation.

MitoPLD, is a member of the phospholipase D superfamily that is anchored on the mitochondrial surface. It has two major functions, one is to generate the lipid messenger phosphatidic acid which is necessary for mitochondrial fusion.^[^ ^41^, ^42^ ^]^ Knockdown of MitoPLD decreases mitochondrial fusion, resulting in mitochondrial fragmentation.^[^ ^43^ ^]^ The other function of MitoPLD is to participate in primary piRNA biogenesis as an endoribonuclease.^[^ ^42^, ^44^, ^45^, ^46^ ^]^ In mice, MitoPLD is highly expressed in testes and in growing oocytes, and loss of *MitoPLD* induces infertility of males but not females.^[^ ^44^, ^47^, ^48^, ^49^, ^50^ ^]^ In *Drosophila*, mutation of *zuc*, an orthologue of mouse *MitoPLD*, leads to female sterility and dorsoventral patterning defects during oogenesis.^[^ ^46^ ^]^ The effect of MitoPLD on females is inconsistent between mice and *Drosophila*.

In this study, we identified *MitoPLD* (referred to hereafter as *pld6*) as a germline‐specific regulator of mitochondrial fusion in zebrafish. The *pld6* mutants exclusively developed into infertile males with no sperm in the testes. Our study further revealed that a germline‐specific MitoPLD‐mediated mitochondrial fusion process is essential for maintenance and differentiation of GSPCs and thus for gonadal development and gametogenesis.

## 2 Results

### 2.1 Mitochondrial Organization Process is Highly Enriched in Juvenile Ovary

**Figure 1:**
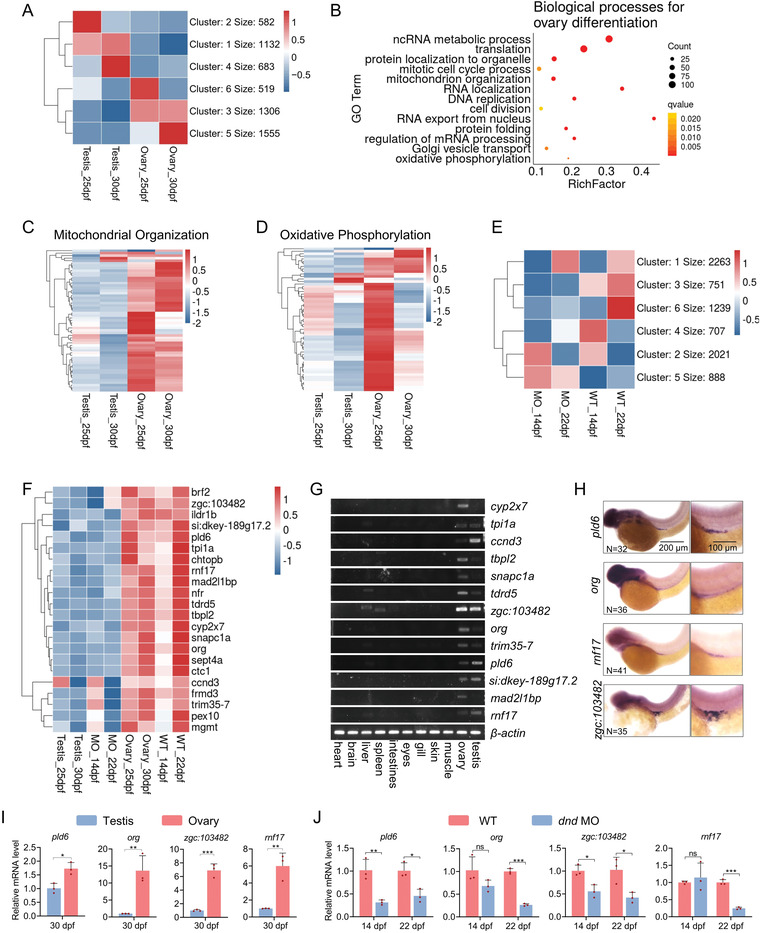
High expression of mitochondrial organization‐related genes in juvenile ovary. A) K‐means clustering of high‐variation genes in gonads. The genes of cluster 5 were highly expressed in the juvenile ovaries at 25 and 30 dpf. B) GO enrichment analysis of cluster 5 genes. C) Heatmap of mitochondrial organization‐related genes expression in juvenile ovary and testis at 25 and 30 dpf. D) Heatmap of oxidative phosphorylation‐related genes expression in juvenile ovary and testis at 25 and 30 dpf. E) K‐means clustering of high‐variation genes in trunks. The expression of cluster 4 genes was lower in *dnd*‐MO than in wildtype at 14 and 22 dpf. F) Heatmap of highly expressed genes overlapped between clusters 5 and 4 from gonads differentiating toward ovary. G) Detection of tissue‐specific gene expression by RT‐PCR. H) Detection of PGC‐specific gene expression by WISH. Scale bar: 200 µm for left; 100 µm for right. *N* represents analyzed embryo number. I) RT‐qPCR verification of differential expression of *pld6*, *org*, *zgc:103482*, and *rnf17* in juvenile ovary and testis of wildtype. Every three gonads were mixed into a sample and three biological replicates were performed. J) RT‐qPCR verification of differential expression of *pld6*, *org*, *zgc:103482*, and *rnf17* in gonads of wildtype and *dnd*‐MO. Every three gonads were mixed into a sample and three biological replicates were performed. The data were expressed as mean ± SD. The *P* values in this figure were calculated by two‐sided *t*‐test. *P < 0.05; **P < 0.01; ***P < 0.001; MO, morpholino; WISH, whole‐mount in situ hybridization; PGC, primordial germ cell; dpf, days post fertilization; RT‐qPCR, reverse‐transcription quantitative PCR.

It is well‐known that the number of GSPCs greatly affects the gonadal differentiation of zebrafish. To better understand the genetic mechanisms underlying this process, we conducted a combined analysis of the RNA‐seq data of juvenile ovaries and testes at 25 and 30 dpf,^[^ ^51^ ^]^ and the microarray data of wildtype and germ cell‐less juvenile gonads at 14 and 22 dpf.^[^ ^52^ ^]^ As to the RNA‐seq data, we conducted cluster analysis and identified 1132 genes from cluster 1, which were highly expressed in putative testes, and 1306 genes from cluster 3, which were highly expressed in putative ovaries (**Figure** **1**A). The testis highly‐expressed genes were significantly enriched in blood vessel development process and actin filament organization process (Figure S1A, Supporting Information), while the ovary highly‐expressed genes were significantly enriched in ncRNA metabolic process, translation process, mitotic cell cycle process, and mitochondrial organization process (Figure 1B). By gene expression matrix analysis, we found that almost all the mitochondrial organization‐related genes were highly expressed in putative ovaries (Figure 1C; Figure S1B, Supporting Information). Moreover, the genes involved in mitochondrial oxidative phosphorylation were also highly expressed in putative ovaries, and the difference was more obvious between the samples at 25 dpf (Figure 1D).

Dnd, as a component of germ plasm, is required for zebrafish germ cell migration and survival,^[^ ^14^ ^]^ and early depletion of PGCs by morpholino knocking down *dnd* promotes testis formation.^[^ ^52^ ^]^ Comparing the transcriptome of wildtype and *dnd*‐knockdown gonads could provide insights on the genetic mechanisms underlying germ cell differentiation. As to the microarray data of wildtype and *dnd* MO‐injected juvenile gonads at 14 and 22 dpf,^[^ ^52^ ^]^ we obtained 888 genes from cluster 5, whose expression was higher in *dnd* MO‐injected gonads and 751 genes from cluster 3, whose expression was higher in the wildtype than in *dnd* MO‐injected gonads at two stages (Figure 1E; Figure S1C, Supporting Information). We further obtained 22 overlapped genes from those genes highly expressed in putative ovaries versus putative testes (1306 genes) and in wildtype versus *dnd* morphants (751 genes). In these overlapped genes, only one mitochondrial organization‐related gene, *pld6*, MitoPLD in zebrafish, was identified (Figure 1F).

To identify the genes which were most likely to be required for gametogenesis and gonadal differentiation, we performed a screening according the following criteria: the genes should be specifically expressed in the gonads at adult stage and in GSPCs at embryonic stage. From 22 genes, we screened 13 which were expressed predominantly in ovary or testis of adults (120 dpf) (Figure 1G). Of these 13 genes, we identified several genes, such as *tdrd5*, *tdrd4* (*rnf17*),^[^ ^16^, ^53^ ^]^ and *org*,^[^ ^54^ ^]^ which were known to play roles in gametogenesis, and *pld6* as well (Figure 1G). We performed reverse transcription PCR (RT‐PCR) analysis and found that 11 genes, except *cyp2x7* and *trim35‐7*, were lowly expressed in *dnd* MO‐injected gonads (Figure 1J; Figure S1F, Supporting Information). Since *rnf17*, *org*, and *zgc:103482* have been reported to be expressed in PGCs,^[^ ^53^, ^54^ ^]^ we explored whether there were novel markers of PGCs by whole‐mount in situ hybridization (WISH) assay. In addition to the above‐mentioned three known genes, the WISH signals of *pld6* were specifically detected in the PGCs of embryos at 3 dpf (Figure 1H; Figure S1G, Supporting Information). We further verified that *pld6*, *org*, *zgc:103482*, and *rnf17* were highly expressed in the juvenile ovaries (Figure 1I). All these results indicated that the mitochondrial organization process might be essential for ovarian differentiation and *pld6* could play a critical role in this process.

### 2.2 *pld6* is Novel Marker for Germ Cells at Different Stages

**Figure 2:**
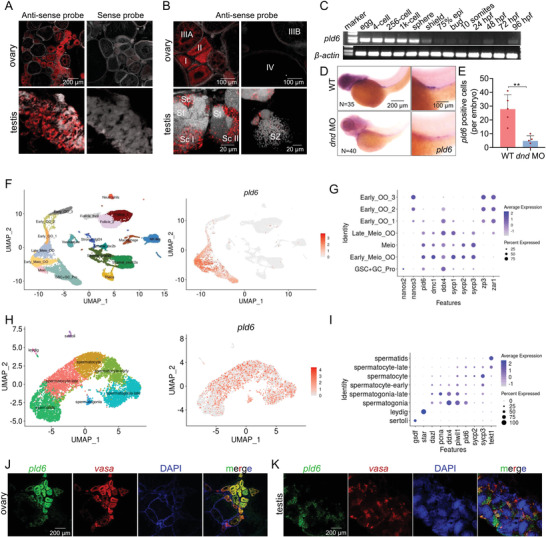
Specific expression of zebrafish *pld6* in germline. A,B) In situ hybridization of ovary and testis cryosections with *pld6* probe. Scale bar: 200 µm for A; 100 µm for B up; 20 µm for B down. C) Expression detection of *pld6* in early embryonic stage by RT‐PCR. D) Expression detection of *pld6* in wildtype and *dnd*‐knockdown 3 dpf embryos by WISH. *N* represents analyzed embryo number. E) Statistical analysis of *pld6*‐positive cells in wildtype and *dnd*‐knockdown 3 dpf embryos in panel D. F) The exhibition of 19 cell types in the ovary at 40 dpf by UMAP (left). The distribution of *pld6*‐expressed cells (right). G) Expression of ten marker genes (*nanos2*, *nanos3*, *pld6*, *dmc1*, *ddx4*, *sycp1*, *sycp2*, *sycp3*, *zp3*, and *zar1*) in the germ cells at different stages. H) The exhibition of 8 cell types in the adult testis by UMAP (left). The distribution of *pld6*‐expressed cells (right). I) Expression of ten marker genes (*gsdf*, *star*, *dazl*, *pcna*, *ddx4*, *piwil1*, *sycp2*, *sycp3* and *tekt1*) in the somatic cells and germ cells at different stages. J) Two‐color fluorescent in situ hybridization of adult ovary with probes of *pld6* and *vasa*. Scale bar: 200 µm. K) Two‐color fluorescent in situ hybridization of adult testis with probes of *pld6* and *vasa*. Scale bar: 200 µm. The data were expressed as mean ± SD. The *P* values in this figure were calculated by two‐sided *t*‐test. **P < 0.01; MO, morpholino; WISH, whole‐mount in situ hybridization; dpf, days post fertilization.

A set of experiments were utilized to analyze the expression of *pld6* during zebrafish gametogenesis. Fluorescence in situ hybridization (FISH) against gonad sections demonstrated that *pld6* mRNA was specifically expressed in oogonia and oocytes (stage I, II, IIIA) in ovary, and spermatogonia in and spermatocytes testis (**Figure** **2**A,B). RT‐qPCR analysis indicated that *pld6* mRNA exhibited a maternal supply and gradually decreased from shield stage (Figure 2C). The *dnd* knockdown specifically eliminated the expression of *pld*6, confirming that *pld6* was specifically expressed in the embryonic PGCs (Figure 2D,E). We further utilized two recently published single‐cell transcriptome data,^[^ ^55^, ^56^ ^]^ to analyze the cell types that express *pld6*. The results showed that, *pld6* were specifically expressed in germ cells including GSCs, early meiotic germ cells, and early oocytes in juvenile ovary (Figure 2F). In detail, *pld6* was co‐expressed with *nanos2* in GSPCs,^[^ ^25^, ^26^ ^]^ and with *sycp1*, *sycp2* and *sycp3* in meiotic germ cells.^[^ ^57^, ^58^, ^59^ ^]^ However, *pld6* was lowly expressed in *zp3‐* and *zar1*‐expressed early oocytes^[^ ^60^, ^61^ ^]^ (Figure 2G). In adult testis, *pld6* was also specifically expressed in germ cells rather than somatic cells (Figure 2H). In detail, *pld6* was highly expressed in spermatogonia and lowly expressed in spermatocytes. Whereas, there was no expression of *pld6* in spermatids (Figure 2I). We then verified the germ cell‐specific expression of *pld6* using two‐color FISH. As seen in Figure 2J,K, both ovary and testis exhibited high expression of *pld6*, which expression signals were colocalized with *vasa* signals, which specifically labeled germ cells. Thus, we identified *pld6*, a gene encoding mitochondrial fusion regulator, as a novel marker for zebrafish germ cells in both sexes.

### 2.3 Depletion of *pld6* Leads to Masculinization and Infertility

**Figure 3:**
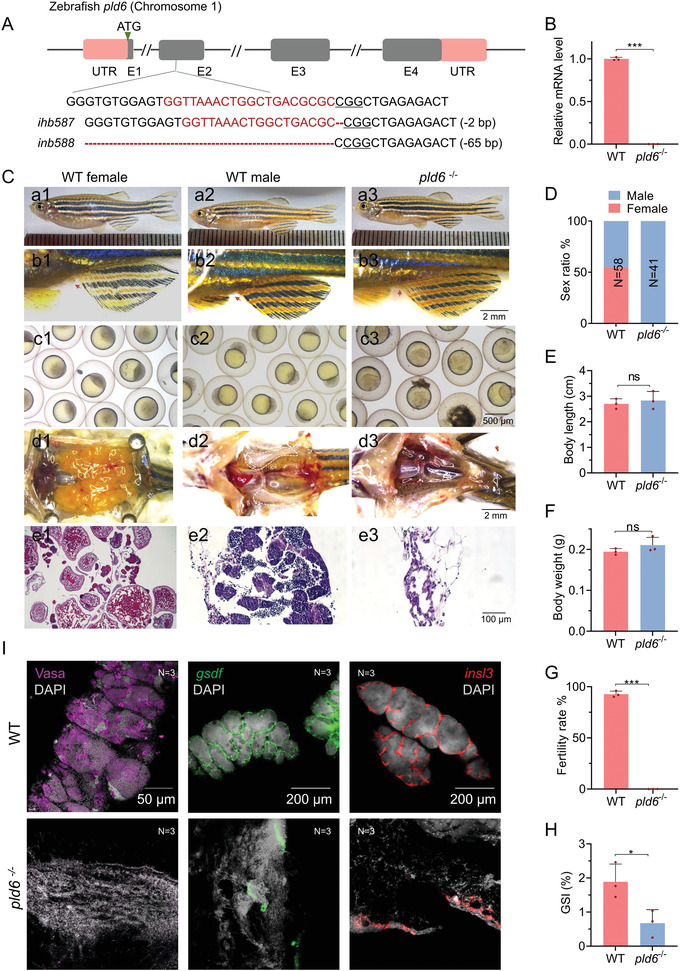
Masculinization and infertility induced by *pld6* deficiency in zebrafish. A) Schematic of zebrafish *pld6* genome locus and the gRNA target. Red font indicates gRNA target sequences. B) RT‐qPCR analysis of *pld6* expression in 22 dpf gonads of *pld6*‐null mutant. C) Morphological and anatomical analyses of wildtype and *pld6*‐null mutant. D) Sex ratio of wildtype and *pld6 ^−/−^* groups. *N* represents analyzed individual number. E,F) Comparison of body length and body weight between wildtype and mutant. G) Comparison of fertilization rates between wildtype and mutant. H) Comparison of the GSI between wildtype males and mutant males. I) Anti‐vasa immunostaining and in situ hybridization of wildtype and mutant testis with probes of *gsdf* and *insl3*. Scale bar: 50 µm for Vasa, 200 µm for *gsdf* and *insl3*. *N* represents analyzed individual number. The data were expressed as mean ± SD. The *P* values in this figure were calculated by two‐sided *t*‐test. *P < 0.05; ***P < 0.001; and ns, no significant difference; RT‐qPCR, reverse‐transcription quantitative PCR; GSI, gonadosomatic index.

To investigate the functions of *pld6*, we generated zebrafish *pld6* mutant by CRISPR/Cas9 approach, and two mutated alleles (*ihb587*, *ihb588*), with deletion of 2 or 65 base pairs, were obtained (**Figure** **3**A). These mutations led to the shift of the open reading frame and the deletion of the conserved PLD domain in the MitoPLD (Figure S2A, Supporting Information). Since there was no phenotypic difference between the homozygous mutants between these two alleles, we only presented the results of *ihb587* allele in subsequent studies. By high resolution melting (HRM) analysis, we identified the homozygotes, the heterozygotes, and the wildtype (Figure S2B, Supporting Information). Transcripts of *pld6* was almost undetectable in the homozygotes (Figure 3B), indicating the effectiveness of the CRISPR‐mediated mutation.

All the *pld6*‐depleted fish were viable, but *pld6*‐null mutant adults were all phenotypically male‐like, judging from subtle golden stripes, slim abdomen shape, and an absence of genital papilla (Figure 3C(a1–a3),(b1–b3),D). Additionally, the *pld6* ^−/−^ mutants displayed body weights and body lengths similar to their wildtype siblings at 3 months post‐fertilization (mpf) (Figure 3E,F). When mated to females, the mutant males exhibited normal sex behavior and induced female spawning. However, no successful fertilization was observed from the mating between wildtype females and the *pld6*‐depleted males (Figure 3C(c1–c3),G). Histological analysis of the gonadal tissues indicated no germ cells in the atrophied testes of the *pld6*‐deficient adults (Figure 3C(d1–d3),(e1–e3)). The gonadosomatic index (GSI) of *pld6* ^−/−^ mutants was significantly lower than that of wildtype males (Figure 3H). To confirm that male infertility defect was induced by genetic loss of *pld6*, we recovered the *pld6* expression by transgenic technology. The results showed that the female individuals were obtained, and the fertility of the mutant was also recovered in the transgenic mutant lines (Figure S2C–E, Supporting Information). No gain of function phenotypes in somatic tissues were detected in the *pld6*‐transgenic fish, probably due to the low expression of *glycerol kinase 5* (*gk5*), which encodes a putative Pld6 interacting factor,^[^ ^62^ ^]^ in somatic tissues instead of gonads (Figure S2F, Supporting Information). Moreover, *gk5* showed high expression in GSCs, which was similar to the expression profile of *pld6* (Figure S2G,H, Supporting Information).

We further identified the cell types in the mutant gonad by examining a set of molecular markers. The expression of Leydig cell‐specific genes *(insl3* and *cyp17a1*), and Sertoli cell‐specific genes (*gsdf* and *amh)* was still detectable in mutant. However, the expression of germ cell‐specific genes, *vasa* and *nanos2*, were not detected in mutant (Figure 3I; Figure S2I, Supporting Information). These results indicated that depletion of *pld6* resulted in the loss of germ cells, but did not affect the development of gonadal somatic cells, finally leading to masculinization and infertility.

### 2.4 Depletion of *pld6* Results in Loss of GSPCs in Juveniles

**Figure 4:**
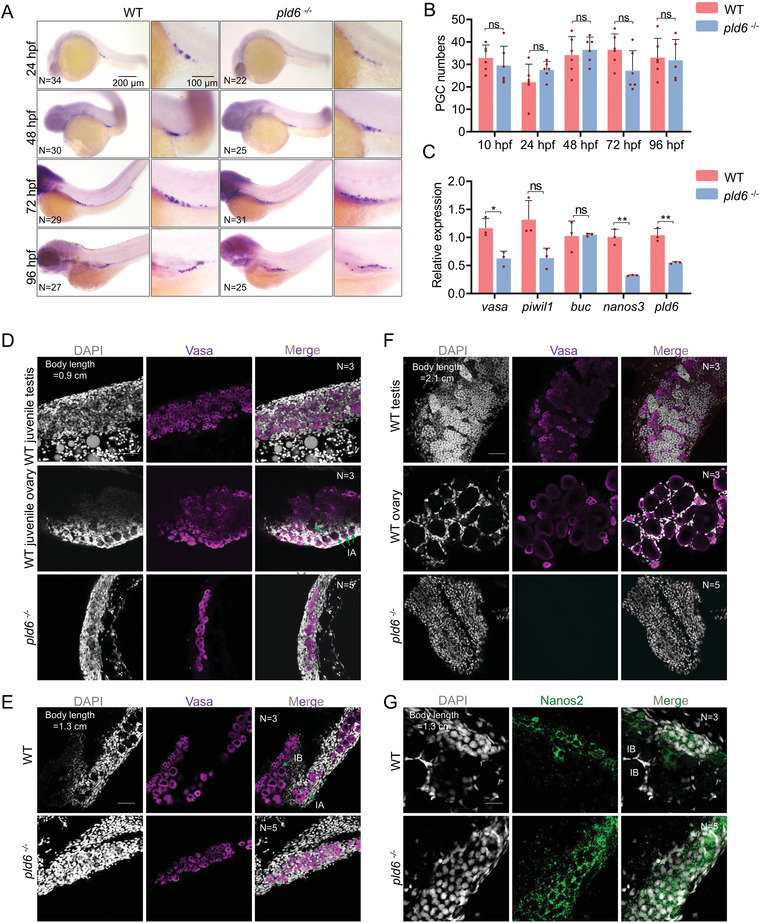
Tracing of gonad development in *pld6* mutant. A) Comparison of PGC numbers between wildtype and mutant by WISH with *vasa* probe. WISH was performed on embryos at 24, 48, 72, and 96 hpf. *N* represents analyzed embryo number. B) Statistical analysis of total PGC numbers in wildtype and *pld6* ^−/−^ embryos. C) RT‐qPCR detection of the expression of germ plasm factors (*vasa*, *piwil1*, *buc*, and *nanos3*) in wildtype and *pld6* ^−/−^ larvae. The decreased expression level of *pld6* was set as positive control. D–F) Tracing of germ cell development in wildtype and *pld6* ^−/‐^ juvenile gonads at 22 dpf (D), 30 dpf (E), and 45 dpf (F). Anti‐vasa staining was performed to label the germ cells, and DAPI staining was performed to label the nuclear. Green arrows marked the stage IA and IB oocytes. *N* represents analyzed individual number. Scale bar: 100 µm. G) Anti‐Nanos2 immunostaining of wildtype and mutant gonads at 30 dpf. *N* represents analyzed individual number. Scale bar: 20 µm. The data were expressed as mean ± SD. The *P* values in this figure were calculated by two‐sided *t*‐test. *P < 0.05; ***P < 0.001; ns, no significant difference; PGC, primordial germ cell; WISH, whole‐mount in situ hybridization; hpf, hours post fertilization; dpf, days post fertilization; RT‐qPCR, reverse‐transcription quantitative PCR.

Zebrafish with PGC depletion have been reported to exclusively develop into infertile males.^[^ ^7^, ^13^, ^52^ ^]^ We wondered whether the infertile phenotype of mutants was due to low number of embryonic PGCs. First, we investigated the formation of PGCs in the *pld6*‐disrupted zebrafish by WISH with *vasa* probe. The PGC number in the null‐mutant was comparable to that in the wildtype (**Figure** **4**A,B). The expression level of germ plasm components such as *piwil1* and *buc* exhibited no difference between the mutant and wildtype (Figure 4C). To determine if the maternally provided *pld6* transcripts were still functional in the early embryonic development stage in the zygotic mutants of *pld6*, we designed morpholino (MO) antisense oligonucleotides to block the maternal expression of *pld6*. As shown in Figure S3A, Supporting Information, the MO specifically and effectively blocked the translation of the fusion reporter, but not the control mRNAs. In the *pld6*‐MO injected embryos at 4‐cell, 4 hpf, 1 dpf, and 2 dpf, the numbers of *vasa*‐positive PGCs were decreased. No obvious difference was observed between wildtype and *pld6‐*overexpressed embryos. Moreover, the decreased PGC numbers in morphants could be rescued by *pld6* mRNA, further demonstrating the specificity of *pld6*‐MO (Figure S3B,C, Supporting Information). These results suggest that maternally provided *pld6* mRNA is required for early PGC formation.

To further understand the phenotype of germ cell‐loss in *pld6^−/−^* adults, we carefully examined the development of germ cells during gonad differentiation. At 14 dpf (juvenile body length = 0.6 ± 0.1 cm), the number of *vasa* positive cells showed no obvious difference between the mutant and wildtype (Figure S3D, Supporting Information). At 22 dpf (juvenile body length = 0.9 ± 0.1 cm), the primitive gonads in wildtype developed into dimorphic gonads, juvenile testis in small size and juvenile ovary in big size (Figure 4D). However, in *pld6*‐deficient mutant, all the gonads were much thinner and smaller than the WT testes, and they only contained small amount of GSPCs (Figure 4D). At 30 dpf (body length = 1.3 ± 0.1 cm), the GSPCs in wildtype was differentiated into stage IA oocytes, and a few stage IB oocytes. However, the germ cells in the mutant failed to differentiate and still stayed in a GSPC‐like state (Figure 4E). At 45 dpf (body length = 2.1 ± 0.1 cm), the gonadal differentiation was completed in wildtype, whereas the germ cells disappeared in the mutant (Figure 4F). To examine the GSPCs in *pld6*‐depleted gonads, we preformed immunofluorescence analysis on 30 dpf gonads against Nanos2, a previously reported GSC marker,^[^ ^25^, ^26^ ^]^ using a recently developed Nanos2 antibody.^[^ ^10^ ^]^ The specificity of the Nanos2 antibody was confirmed by staining with a *nanos2^−/−^* mutant gonad and an SSC‐increased *cyp11a2^−/−^* mutant gonad (Figure S4, Supporting Information). In wildtype, the differentiated stage IB oocytes were observed and a few Nanos2‐positive GSCs were distributed in the edge of gonads. While, in mutants, an increased number of Nanos2‐positive cells were detected throughout the gonads (Figure 4G). Therefore, in the *pld6* mutants, the GSPCs retained and failed to differentiate into early oocytes in 30 dpf gonads, and they gradually disappeared during 30 to 45 dpf. The absence of germ cells in the mutant gonads led to female‐to‐male sex reversal at the juvenile stage and infertility of males at the adult stage. All these results indicate that *pld6* is necessary for differentiation and survival of GSPCs during gonadal development, and thus is required for female differentiation and proper gametogenesis.

### 2.5 *Pld6* Depletion Inhibits GSPC Proliferation and Differentiation and Results in GSPC Apoptosis

**Figure 5:**
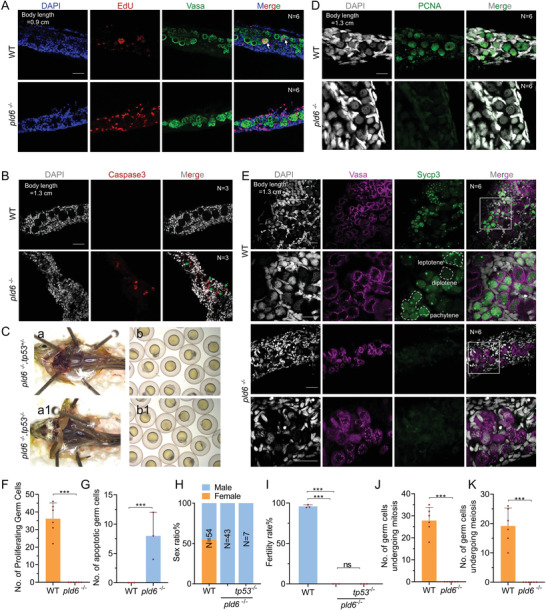
Blocking of germ cell self‐renewal and differentiation in *pld6*‐ depleted gonad. A) EdU staining of proliferating cells in wildtype and *pld6* ^−/‐^ juvenile gonads. The germ cells were marked by anti‐vasa immunostaining. *N* represents analyzed individual number. Scale bar: 100 µm. B) Apoptosis detection in wildtype and *pld6* ^−/−^ juvenile gonads by anti‐Caspase3 immunostaining. *N* represents analyzed individual number. Scale bar: 100 µm. C)Morphological observation of gonads and offspring of *pld6* ^−/−^ and *pld6* ^−/−^; *tp53^−/−^* double mutant. D) Detection of mitosis in wildtype and *pld6* ^−/‐^ juvenile gonads by anti‐Pcna immunostaining. *N* represents analyzed individual number. Scale bar: 100 µm. E) Detection of meiosis in wildtype and *pld6* ^−/−^ juvenile gonads by anti‐Sycp3 immunostaining. *N* represents analyzed individual number. Scale bar: 100 µm. F) Statistical analysis of total number of proliferating germ cells. G) Statistical analysis of the total number of apoptotic germ cells. H) Sex ratio of wildtype, *pld6^−/−^*, and *pld6* ^−/−^; *tp53^−/−^* double mutant. I) Fertilization rates of wildtype, *pld6^−/−^* and *pld6* ^−/−^; *tp53^−/−^* double mutant. J) Statistical analysis of the total number of germ cells undergoing mitosis. K) Statistical analysis of the total number of germ cells undergoing meiosis. The data were expressed as mean ± SD. The *P* values in this figure were calculated by two‐sided *t*‐test. ***P < 0.001; ns, no significant difference.

To explore the molecular mechanism underlying the loss of germ cells in *pld6* ^−/−^ gonads, we further analyzed the proliferation and apoptosis of GSPCs. By EdU incorporation assay in combination with co‐immunostaining with Vasa, we found high numbers of EdU‐positive cells in both wildtype and mutant gonads at 22 dpf. In wildtype, most EdU‐positive cells were perfectly co‐localized with Vasa‐positive cells. In contrast, no EdU‐positive cells showed co‐localization with Vasa‐positive cells in mutant (**Figure** **5**A,F). These indicated that the depletion of *pld6* only led to proliferation defects of GSPCs. In the immunoassay with Caspase3, we detected more apoptotic germ cells in the mutant gonad than in wildtype gonad at 22 dpf (Figure 5B,G). To determine whether the loss of germ cells in *pld6* mutant could be rescued by blocking apoptotic pathway, we induced a *tp53* mutation into the *pld6* mutant, since tumor protein Tp53 is an important activator of apoptosis.^[^ ^63^ ^]^ In contrast to previous reports of other germ cell‐less mutants,^[^ ^63^, ^64^ ^]^ double homozygotes (*pld6* ^−/−^; *tp53* ^−/−^) still exhibited a sterile‐male phenotype, resembling that of single *pld6* mutant (Figure 5C,H,I). This indicates that the loss of germ cells in *pld6* mutant was independent of the Tp53‐mediated apoptotic pathway.

We then examined whether *pld6* was required for mitotic proliferation and meiosis of germ cells in the bipotential gonad of juveniles. We investigated the cell proliferation by immunofluorescence with a mitosis marker Pcna^[^ ^65^ ^]^ in the *pld6* mutant gonad at 22 dpf, and found that there was no Pcna‐positive mitotic cell in the mutant gonad (Figure 5D,J). Subsequently, we examined the differentiation of GSPCs in the *pld6*‐mutated gonads by a meiosis marker Sycp3.^[^ ^65^ ^]^ The results showed that Sycp3‐positive meiotic cells at different stages, such as leptotene, pachytene, and diplotene stages, were enriched in the wildtype gonad (Figure 5E,K). In contrast, Sycp3 was not expressed in all the Vasa‐positive cells of *pld6*‐deficient gonad (Figure 5E,K), indicating that the accumulated germ cells were premeiotic GSPCs. Therefore, it was concluded that the GSPCs in the *pld6*‐mutants did not possess mitosis or meiosis, and they entered apoptosis in a Tp53‐independent way.

### 2.6 *Pld6* Mutation Disrupts Mitochondrial Homeostasis and piRNA Biosynthesis in Gonad

**Figure 6:**
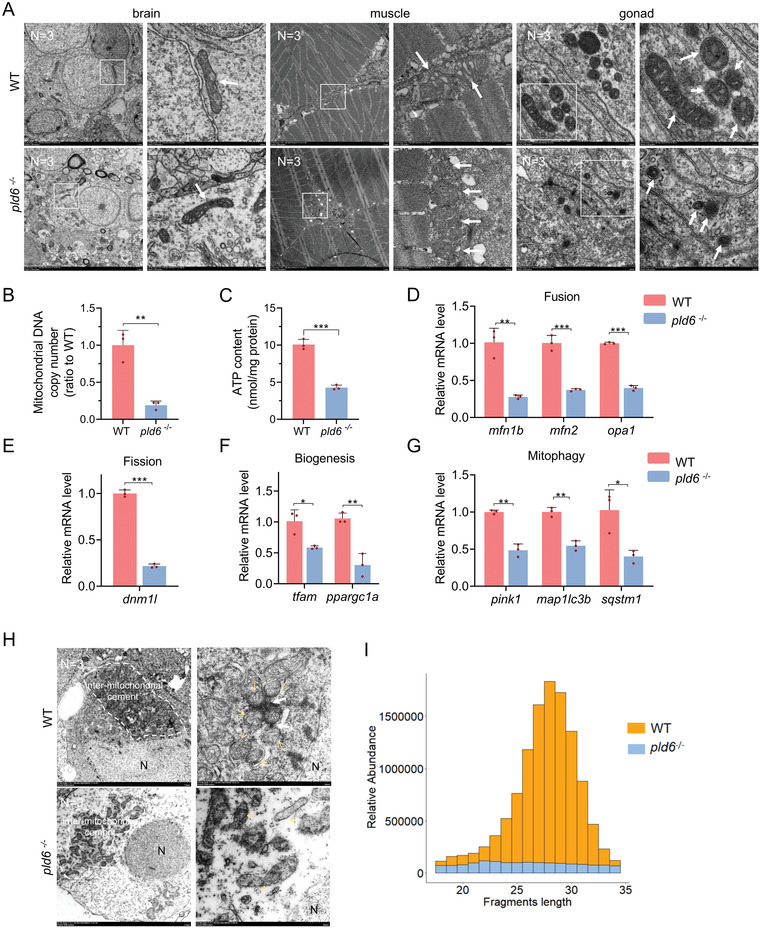
Imbalance of mitochondrial homeostasis and disruption of piRNA biogenesis in *pld6*‐deficient gonad. A) Mitochondria morphological observation of brain, muscle, and juvenile gonad in wildtype and *pld6* ^−/‐^ by the TEM. The box indicates the magnified area. The arrow points at mitochondria. N represents analyzed individual number. B) Comparison of mtDNA copy numbers between wildtype and *pld6* ^−/‐^ juvenile gonads. Every three gonads were mixed into a sample and three biological replicates were performed. C) Comparison of ATP content between wildtype and *pld6* ^−/‐^ juvenile gonads. Every three gonads were mixed into a sample and three biological replicates were performed. (D)RT‐qPCR detection of expressions of mitochondrial fusion factors (*mfn1*, *mfn2* and *opa1*). E) RT‐qPCR detection of expression of mitochondrial fission factors (*dnm1l*). F) RT‐qPCR detection of expressions of mitochondrial biogenesis factors (*tfam* and *ppargc1a*). G) RT‐qPCR detection of expressions of mitochondrial mitophagy factors (*pink1*, *map1lc3b*, and *sqstm1*). H) TEM observation of aggregation of mitochondria around the nucleus and nuage formation in wildtype and *pld6* ^−/−^ juvenile gonads. The dotted line indicates the inter‐mitochondrial cement. The yellow arrow points at the mitochondria. The white arrow points at the electron‐dense nuage. *N* represents analyzed individual number. I) Fragment length distribution of sequences cloned from wildtype and *pld6* ^−/−^ mutant gonads at 22 dpf. piRNAs (26–31 nt) represent approximately 71.97% of the small RNA species between 18 and 34 nt in length in wildtype and only 36.48% in *pld6* ^−/−^ mutant. The data were expressed as mean ± SD. The *P* values in this figure were calculated by two‐sided *t*‐test. *P < 0.05; **P <0.01; ***P <0.001; TEM, transmission electron microscope; RT‐qPCR, reverse‐transcription quantitative PCR.

In mammals and fly, the orthologues of *pld6*, such as mouse *mitoPLD* and *Drosophila zuc*, have been reported to be necessary for mitochondrial fusion,^[^ ^66^, ^67^ ^]^ and the balance between fission and fusion is an important mechanism underlying the control of mitochondria number and size. In this study, we investigated the mitochondria morphology and structure in *pld6*‐null mutant. Since the morphology of mitochondrial networks is highly complex and diverse in different cell types, we carefully compared mitochondria networks in brain, muscle, and gonad of juveniles at 22 dpf between mutant and wildtype. By transmission electron microscopy, we observed that in brain and muscle, mitochondrial morphology of mutant looked similar to that of wildtype (**Figure** **6**A). Significant differences were found only in the gonads. The mitochondria in the wildtype were round or tubular with obvious mitochondrial cristae, whereas the mitochondria in mutant were condensed with smaller size, higher density, and unclear cristae (Figure 6A). In accordance with this, by analyzing the WT gonads and the mutant gonads at 22 dpf, we found that the mitochondrial number and intracellular ATP in the mutant was significantly decreased (Figure 6B,C).

To further investigate the effect of *pld6* depletion on the mitochondrial homeostasis, we analyzed mitochondrial dynamics (fusion and fission),^[^ ^35^, ^68^ ^]^ biogenesis,^[^ ^69^, ^70^ ^]^ and mitophagy^[^ ^71^, ^72^ ^]^ in the gonad of juveniles at 22 dpf. The expression of several genes related to mitochondrial fusion, such as *mfn1b* and *mfn2* (encoding the GTPases responsible for mitochondrial fusion) and *opa1* (interacting with *mfn* to regulate fusion), were significantly lower in *pld6* ^−/−^ than in wildtype (Figure 6D). The expression of *dnm1l* (zebrafish orthologue of mouse *Drp1*) involved in mitochondrial fission was also downregulated in the mutant (Figure 6E). Nuclear‐encoded transcription factors, *tfam* (*mitochondrial transcription factor A*) and *ppargc1a* (*peroxisome proliferator activated receptor gamma co‐activator 1 alpha*) are the master regulators of mitochondrial biogenesis. In *pld6* ^−/−^ mutant, the expression of *tfam* and *ppargc1a* were decreased, indicating that the *de novo* mitochondrial synthesis was impaired (Figure 6F). The damaged mitochondria are mainly cleared by mitophagy, and excessive or untimely fission or fusion may be detrimental to mitophagy.^[^ ^72^, ^73^ ^]^ As seen in Figure 6G, the expression of main factors mediating mitophagy (encoded by *pink1*, *map1lc3b*, and *sqstm1*) were lower in the mutant than in wildtype. Moreover, we analyzed the expression cell types of these genes related to mitochondrial physiology using scRNA‐seq.^[^ ^55^, ^56^ ^]^ Unlike the expression pattern of *pld6*, the other fusion regulators *mfn2* and *opa1* are expressed not only in germ cells but also in somatic cells such as follicle cells, theca cells, and immune cells in ovary (Figure 2F; Figure S4A, Supporting Information), and Sertoli cell and Leydig cell in testis (Figure 2H; Figure S4B, Supporting Information). These results suggested that *pld6*‐deficiency resulted in the imbalance between mitochondrial fission and fusion, thus influencing the number, morphology, function of mitochondria of GSPCs in differentiating gonads. Given that mitochondrial homeostasis is essential for cell survival and cell fate determination, the defects of mitochondrial organization and function in the *pld6* mutant GSPCs would result in the defects of proliferation, differentiation, and survival of GSPCs.

Nuages, known as the pi‐bodies in zebrafish, exist in the form of the cementing material between mitochondria (inter‐mitochondrial cement) of GSPCs.^[^ ^74^ ^]^ We further detected the formation of this nuage structure in *pld6* ^−/−^ juvenile gonads at 22 dpf. TEM showed that mitochondrial clusters were detected at a particular region adjacent to the nucleus in both wildtype and *pld6* ^−/−^ GSPCs (Figure 6H, up), but the electron‐dense pi‐bodies between mitochondria were absent in *pld6* ^−/−^ (Figure 6H, down), suggesting that the formation of the nuage was impaired in mutant. Nuage is generally considered to be the center for piRNA biogenesis and Pld6 has been reported to have phosphodiesterase and/or ribonuclease activity in piRNA maturation.^[^ ^42^, ^44^, ^45^, ^67^, ^75^, ^76^ ^]^ To evaluate the impact of the *pld6* mutation on piRNA biogenesis, we deep‐sequenced 20–33 nt total small RNAs obtained from wildtype and *pld6* ^−/−^ gonads at 22 dpf. Based on the total miRNA level, total small RNAs were normalized.^[^ ^48^ ^]^ We analyzed the size distribution of the sequences and found that the piRNA peak within 26–31 nt was much lower in *pld6* ^−/−^ juvenile gonad than in wildtype (Figure 6I). Since piRNA display the characteristics of 5′ Uridine preference (1U) and a 10th nucleotide adenosine bias (10A), we next examined the base composition at position 1 and position 10 in mutant. As shown in Figure S6A,B, Supporting Information, piRNA in *pld6* ^−/−^ gonad exhibited the marked reduction in 1U and 10A. The major function of piRNA is to repress transposons to maintain genome integrity and germ cells survival. We further investigated the transposon expression levels. As expected, we observed obvious increased levels of several transposon elements such as long terminal repeats (LTRs), non‐LTRs, and DNA elements in *pld6* ^−/−^ germ cells in juvenile gonad at 22 dpf (Figure S6C, Supporting Information). These results suggested that loss of piRNA, the defenders of the genome, induced transposon activation in *pld6*‐deficient germ cells.

## 3 Discussion

**Figure 7:**
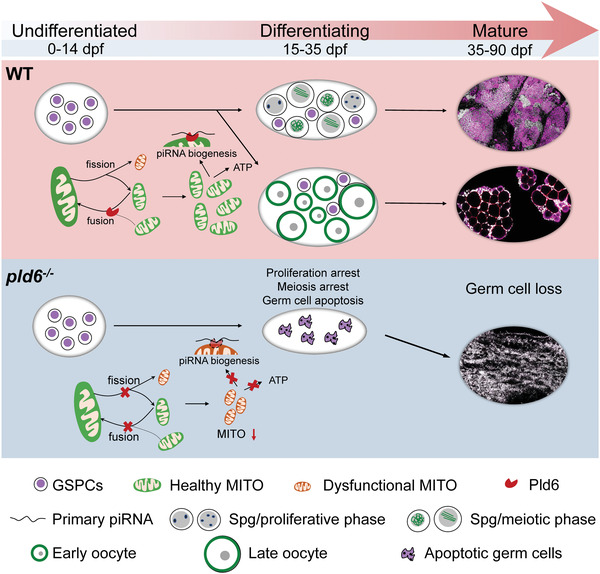
A graphic summary of the role of *pld6* in germ cell development. During zebrafish sex differentiation, Pld6 regulates the mitochondrial fusion and fission in germ cells, leading to the sufficiency of energy supply and the stabilization of piRNA biosynthesis. In wildtype, *pld6* safeguards the health of mitochondria and facilitates the generation of sufficient mitochondria in GSPCs thus promotes the differentiation of juvenile gonads and the maturation of ovary and testis. In mutant, loss of *pld6* leads to the scanty amount and dysfunctional mitochondria in GSPCs, further leads to the germ cell apoptosis, and arrest of meiosis and proliferation during gonad differentiation. Therefore, a GSPCs‐specific *pld6* mediate‐mitochondrial fusion and piRNA biogenesis promotes survival, proliferation, and differentiation of GSPCs.

Knowledge of the molecular mechanisms governing GSPC development and sex differentiation are still in the infancy stage. In the present study, we identify *pld6* as a novel marker of germ cells, and reveal that *pld6*‐mediated mitochondrial fusion is required for maintenance and differentiation of GSPCs in zebrafish. Our study indicates that the insufficiency and dysfunction of mitochondria in bipotential juvenile gonads lead to the proliferation and differentiation failures of GSPCs and germ cell loss of the *pld6* mutant (**Figure** **7**).

Germplasm components are essential for PGC formation and a majority of germplasm components are found to have germline‐specific expression.^[^ ^14^, ^15^, ^16^, ^27^, ^28^, ^77^, ^78^, ^79^ ^]^ In this study, we identified 13 genes specifically expressed in gonad, including a novel germline‐specific gene, *pld6*, encoding the regulator of mitochondrial fusion (Figure 1G). In detail, zebrafish *pld6* showed strong maternal supply and PGC‐specific characteristics at 3 dpf, and *pld6* was continuously expressed in germ cells, especially in GSCs, from juvenile to adult stage (Figure 2). Knockdown of *pld6* reduced the PGC number, but did not induce any other developmental defects at early embryonic stage (Figure S3A,B, Supporting Information), whereas zygotic knockout of *pld6* resulted in female‐to‐male sex reversal and male infertility (Figure 3). Our finding of *pld6* mutant was quite different from the phenotypic changes induced by the disruption of the other two mitochondrial fusion‐regulating factors, that is, *opa1* knockdown,^[^ ^80^ ^]^ and *mfn2* knockdown or mutation^[^ ^81^, ^82^ ^]^ in zebrafish. In detail, *opa1* knockdown caused abnormal blood circulation and heart defects in embryonic development,^[^ ^80^ ^]^ and knockdown of zebrafish *mfn2* causes morphological and motility defects.^[^ ^81^ ^]^ Analysis of their functions in germline was prevented as these two morphants did not survive past 7 dpf. Unlike morphants, mutation of *mfn2* developed normally but subsequently manifested a progressive motor dysfunction and pathological alterations to the neuro‐muscular junction.^[^ ^82^ ^]^ The *mfn2* mutants were not able to breed successfully, indicating that *mfn2* also plays a role in the reproduction progress of zebrafish.^[^ ^82^ ^]^ Such phenotypic differences might be due to the different spatiotemporal expressions of these genes. Unlike the PGC‐specific expression of *pld6*, *opa1*, and *mfn2* were ubiquitously expressed at embryonic stage.^[^ ^80^, ^81^ ^]^ In gonad tissues, expression of *pld6* was enriched in germ cells (Figure 2), whereas, *opa1* and *mfn2* were expressed not only in germ cells but also in somatic gonadal cells. The transcripts of *opa1* and *mfn2* were also detected in immune cells and vasculature cells (Figure 2F,H; Figure S5A,B, Supporting Information). To avoid embryonic lethal and secondary effects, a recently established germline‐specific knockout approach will be helpful for revealing function of these mitochondrial fusion factors in germ cell development.^[^ ^83^ ^]^

Previous studies obtained inconsistent results on whether mitochondrial fusion is dispensable for self‐renewal of undifferentiated stem cells. Although mitochondrial fusion is essential for male fertility and reproduction in both *Drosophila* and mice, and its effect on GSCs is different between these two species.^[^ ^37^, ^38^, ^84^, ^85^, ^86^ ^]^ In *Drosophila*, depletion of *Mfn* or *Opa1* in male germline resulted in a substantial loss of GSCs.^[^ ^37^ ^]^ In mice, depletion of *Mfn1* and *Mfn2* induced loss of all differentiating germ cells in males, but it did not affect the proliferation of stem‐like undifferentiated spermatogonia.^[^ ^85^ ^]^ In our work, both GSPCs and differentiating germ cells were lost with *pld6* depletion, and *pld6*‐deficient GSPCs failed to proliferate through mitosis and to differentiate through meiosis. Thus, our study demonstrates that a germline‐specific mitochondrial fusion machinery, mediated by Pld6, is required for both maintenance and differentiation of GSPCs (Figure 5).

MitoPLD, which is the ortholog of zebrafish *pld6*, is also known as Zucchini (Zuc), and it is a candidate ribonuclease participating in primary piRNA biogenesis.^[^ ^46^, ^75^, ^87^ ^]^ Mouse *MitoPLD* is predominantly expressed in the testis and growing oocytes, which is in line with the expression characteristics of zebrafish *pld6*. The *MitoPLD^−/−^* female mice showed no obvious phenotypic change, and male *MitoPLD* mutant mice displayed meiotic arrest during spermatogenesis and defects in piRNA generation.^[^ ^48^, ^49^ ^]^ In consistent with the observations in mice, we found meiosis failure of GSCs and the loss of piRNA clusters in zebrafish (Figures 5 and 6). However, there exists great differences in the arrested stage of the meiotic prophase and the adult gonad cell types between mouse *MitoPLD* mutants and zebrafish *pld6* mutants. In our study, meiosis initiation did not occur during gonad differentiation and all the germ cells were lost in *pld6*‐depleted testis, while, spermatocytes were arrested at the zygotene stage, and undifferentiated spermatogonia were still observed in *MitoPLD^−/−^* mice.^[^ ^48^ ^]^ These observations suggest that MitoPLD plays a conserved role in the piRNA biogenesis, but it plays different functions in regulating meiosis process and germ cell survival between fish and mammal.

The germ cell‐less and sterile male phenotype in our study is consistent with the defects induced by the mutation of piRNA production‐related genes including *ziwi*,^[^ ^28^ ^]^ *zili*,^[^ ^77^ ^]^ and *vasa* ^[^ ^27^ ^]^ in zebrafish. However, the mechanism underlying the germ cell death in these mutants is different. Apoptosis is the most common reason whereby the germ cell is lost in zebrafish.^[^ ^88^ ^]^ The phenotype of *zili* and *vasa* mutant is not associated with canonical apoptosis, while, the phenotype of *ziwi* and *pld6* mutant centers on apoptosis in the bipotential gonad, as judged by observation of the hyperactivated Caspase‐3 apoptosis signaling. However, *tp53* depletion did not rescue germ cell‐less phenotype in the *pld6* ^−/−^ mutant (Figure 5B,C). Based on these results, it is proposed that germ cell death induced by piRNA loss might be independent of p53‐mediated apoptotic pathway.

In summary, our study reveals that a germline‐specific regulator of mitochondrial fusion, MitoPLD, is essential for the maintenance and differentiation of GSPCs. Furthermore, the over‐fragmented mitochondria and the disrupted nuage structure in *pld6* mutant zebrafish GSPCs further confirm the relationship between mitochondria fusion and piRNA biogenesis.

## 4 Experimental Section

### Contact for Reagent and Resource Sharing

Further information can be available and requests for resources and reagents will be fulfilled by the Lead Contact, Yonghua Sun (yhsun@ihb.ac.cn)

### Fish Lines Availability

The *pld6* mutant lines were generated by authors. The mutant lines were submitted and deposited in China Zebrafish Resource Center of the National Aquatic Biological Resource Center (CZRC‐NABRC, Wuhan, China, http://zfish.cn).

### Data Availability

RNA‐seq raw data, processed expression matrix, sample information were stored at GEO (https://www.ncbi.nlm.nih.gov/geo/) and SRA (https://www.ncbi.nlm.nih.gov/sra) under accession number of GSE175979,^[^ ^51^ ^]^ GSE57046,^[^ ^52^ ^]^ GSE191137,^[^ ^55^ ^]^ and PRJNA807002 (piRNA data), and National Genomics Data Center (CNCB‐NGDC, https://bigd.big.ac.cn/gsa, CRA003925.^[^ ^56^ ^]^ The code of all analysis steps was available at a repository named Yichel518/MitoPLD‐analysis from Github (https://github.com/).

### Zebrafish Maintenance

All the zebrafish lines used in this study were AB and raised in the China Zebrafish Resource Center of the National Aquatic Biological Resource Center (CZRC‐NABRC, Wuhan, China, http://zfish.cn). The embryos were staged according to morphology, as previously described.^[^ ^89^, ^90^ ^]^ The zebrafish experiments were performed under the approval of the Institutional Animal Care and Use Committee of the Institute of Hydrobiology, Chinese Academy of Sciences under protocol number IHB2014‐006.

### Generation of pld6 Mutants By CRISPR/Cas9

The *pld6* mutants were generated by CRISPR/Cas9‐mediated mutagenesis.^[^ ^91^ ^]^ The target sites were designed using an online tool (http://www.crisprscan.org/). The gRNA target containing PAM sequence (underlined) of *pld6* was 5′‐TTAAACTGGCTGACGCGCCGG‐3′. pT3TS‐zCas9 was used for cas9 mRNA transcription, and capped Cas9 mRNA was generated using T3 mMESSAGE Machine kit (AM1344, Ambion, Austin, Texas). gRNA was generated using in vitro transcription with T7 RNA polymerase (P2075, Promega, Madison, Wisconsin). Cas9 mRNA and gRNA were co‐injected into wildtype embryos at one‐cell stage. The DNA fragment covering the target regions of *pld6* was amplified using the primer pairs listed in Table S1, Supporting Information. The PCR products were subjected to the Sanger sequencing to evaluate the efficiency of mutagenesis or to identify the genotypes of the mutants.

### cDNA Synthesis and Reverse‐transcription Quantitative PCR (RT‐qPCR)

Total RNA was isolated from gonads or embryos of wildtype and *pld6^−/−^* using tRizol (Invitrogen). cDNA was synthesized using an oligo‐dT primer and RevertAid First Strand cDNA Synthesis Kit (Thermo Fisher Scientific). RT‐qPCR was performed using the SYBRGreen Supermix from BioRad (USA) on a BioRad CFX96.^[^ ^92^ ^]^ The samples were tested in biological triplicates for each gene, and resultant *C*q values were averaged. Data were processed using 2^−ΔΔ^ *^C^* ^q^ method.^[^ ^93^ ^]^ All gene‐specific primers used for RT‐qPCR were listed in Table S2, Supporting Information.

### In Situ Hybridization and Counting of PGCs

For whole‐mount in situ hybridization (WISH), embryos or juvenile gonads were collected and fixed at the certain time points. WISH was performed with the following probes: *pld6*, *vasa*,^[^ ^94^ ^]^ *insl3*,^[^ ^95^ ^]^ and *gsdf*,^[^ ^96^ ^]^ as previously described.^[^ ^97^ ^]^ For counting of PGCs, embryos hybridized with *vasa* probe were mounted in 100% glycerin and acquired of images using a 6.3× objective (Leica). PGC numbers at 24, 48, 72, and 96 hpf (Figure 4A,B; Figure S3B,C, Supporting Information) were counted two‐sides under the microscopes (Leica Z16 APO). For in situ hybridization of frozen sections, adult gonads were stripped, embedded, and then sectioned (10 µm). The ISH was performed, as previously described.^[^ ^98^ ^]^ Briefly, the slices were fixed in 4% paraformaldehyde for 15 min at room temperature, and then washed three times in PBS for 5 min each. The probes were incubated at 70 °C overnight. Photographs were taken using a laser‐scanning confocal inverted microscope (SP8, Leica) with an LD C‐Apo 40 × /NA 1.1 water objective.

### High‐Resolution Melting Curve Analysis

Polymerase chain reaction (PCR) and melting curve analysis were performed to identify genotypes by previously reported method.^[^ ^99^ ^]^ Briefly, the 20 µL of PCR reaction system consisted of 0.5 µL Eva green dye (Biotium, USA), 10 µL of 2 × Taq Master Mix (Vazyme, China), 0.5 µL of each primer (10 µm) (5′‐ TGTTGCGTTTGTTCTGGG ‐3′ and 5′‐ GACGCAGACTTGAGGTGAA ‐3′), 2 µL of genomic DNA (100 ng µL^−1^), and water (as remaining part). PCR was conducted as follows: 95 °C for 3 min, then 34 cycles of 95 °C for 10 s, 60 °C for 30 s. After the final step, the plate was heated to 65 °C and maintained for 5 s, and then heated up to 95 °C at a rate of 0.2 °C per second, and rapidly cooled to 4 °C. Melting curves were generated with a LightScanner HR 96 (Idaho Technology) within the range of 65 to 95 °C and analyzed with Bio‐Rad CFX Manager 3.1 software (Bio‐Rad Laboratories, Inc.).

### Histological Analysis

Wildtype fish and *pld6^−/−^* mutant at specific developmental stage were collected for histological analysis. After anesthesia, the intact gonadal tissues were anatomized from wildtype and *pld6^−/‐^* at 3 mpf to obtain the GSI, and GSI was calculated as gonad weight divided by body weight (percentage). The gonad was fixed in 4% PFA (Sigma, St. Louis, MO) overnight at 4 °C and embedded in paraffin. Then the sections (7 µm) were stained with hematoxylin and eosin (H&E) and photographed under a microscope (Olympus BX53).

### Immunostaining of Gonads

Immunofluorescence staining was performed according to the previously described procedures.^[^ ^100^ ^]^ The following primary antibodies were used for immunofluorescence staining: rabbit anti‐Vasa (1:200, Gentex, RRID: AB_2847856, https://scicrunch.org/resolver/AB_2847856), rabbit anti‐Nanos2 (1:200, sunlab_052, RRID:AB_2895084, http://antibodyregistry.org/AB_2895084), rabbit anti‐Caspase3 (1:200, Boster Biological Technology, RRID: AB_2890203, http://antibodyregistry.org/AB_2890203), rabbit anti‐Pcna (1:1000, Aviva Systems Biology, RRID: AB_841619, http://antibodyregistry.org/AB_841619), and rabbit anti‐Sycp3 (1:200, Abcam, RRID:AB_2895074, https://antibodyregistry.org/AB_2895074). For Nanos2 antibody, His‐tagged fusion proteins containing full‐length of zebrafish Nanos2 was generated in E. coli and was purified by Ni resin (BeyoGold) and used for immunization. A polyclonal antibody (anti‐Nanos2) was generated by immunizing rabbits with the His‐tagged protein for five times. The antibody was purified using an affinity column that was covalently conjugated to the antigen. Anti‐rabbit Alexa Fluor 488 (1:500, Molecular Probes, RRID:AB_2535792, https://scicrunch.org/resolver/AB_2535792) and Anti‐rabbit Alexa Fluor 680 (1:500, Molecular Probes, RRID: AB_2535736, http://antibodyregistry.org/AB_2535736) were used as secondary antibodies. To label nuclear, gonads were counterstained with 1 µg mL^−1^ DAPI at 4 °C overnight. Immunostained gonad tissues were washed, cleared, and mounted in 75% antifade mounting medium (1 × PBS, 75% glycerol, 2% *n*‐propyl gallate) for imaging on a Leica SP8 confocal microscope with an LD C‐Apo 40 × /NA 1.1 water objective. The percentage of positive signals was quantified as the proportion of the area of positive signals to the total area of DAPI in each field. The area was measured by Image J. A series of sections were obtained from at least three individual zebrafish for image analysis.

### Morpholino Oligo Injection and Efficiency Test

Morpholino oligo of *pld6* was obtained from Gene Tools (Philomath, Oregon). The sequence of the MO is: 5′‐ TGAACACGTCCATCGAGATGACAAT ‐3′ (translation‐blocking MO covering the translation initiation start, underlined). Amount of MO injected in this study was 2 ng per embryo. To analyze the specificity and efficacy of the translation blocking MO, pCS2+‐gfp‐reporter plasmid was created which harbor the respective morpholino target sequence, fused in frame to the GFP ORF. The gfp‐reporter mRNA was co‐injected into one‐cell stage embryos in combination with the targeting morpholino. A non‐reporter *gfp* mRNA was also co‐injected with *pld6* MO which was used as negative control and *mCherry* mRNA was used as injection indicator. At sphere stages, embryos were assayed for GFP and mCherry fluorescence.

### EdU Assay

Wildtype or *pld6^−/−^* juveniles at 22 dpf were first incubated with 400 µm EdU for 24 h, and then their gonads were dissected and fixed in 4% PFA overnight at 4 °C. After removing the 4% PFA, the gonads were neutralized with 2 mg mL^−1^ glycine solution at room temperature for 5 min, and then washed with 3% BSA/PBS twice. Subsequently, the infiltration of gonads was enhanced with 1% Triton X‐100/PBS for about 2 h at room temperature. Finally, the signaling of cell proliferation was detected with a Yefluor 594 EdU Imaging Kit (40276ES76, YEASEN, China) according to the manufacturer's instruction.

### Transmission Electron Microscope Observation of Mitochondria

Brain, muscle, and gonad tissues from 22 dpf wildtype or *pld6^−/−^* were collected and fixed with 100 µL 2.5% glutaraldehyde at 4 °C overnight. The tissue samples were embedded in Epon812 resin and cut into 70–80 nm slides with an ultrathin slicer after dehydration. The samples were then stained with uranium acetate and lead citrate and observed under TEM (Hitachi, HT7700).

### Analysis of RNA‐Seq and Microarray Data

First, FastqC (0.11.8) and MultiQC (0.9)^[^ ^101^ ^]^ were used to evaluate the raw read quality. Reads were mapped to the corresponding reference genome (Ensembl 96) using Hisat2 (2.0.5)^[^ ^102^ ^]^ with the parameters of “– dta‐x – rna‐strandness RF” and “– known spliceset‐infile”. The data were converted into BAM format by Hisat2, and BAM files were sorted by Samtools (1.5).^[^ ^103^ ^]^ The duplicated sequence was removed from BAM files using the Picard (2.18.15) MarkDuplicates tool. BAM files were counted by HTSeq (0.9.1)^[^ ^104^ ^]^ with the parameters of “‐t exon ‐i gene_id ‐r pos ‐s reverse”. The expression matrix was standardized by TMM using R package edgeR (3.30.3).^[^ ^105^ ^]^ Finally, cor function and Spearman's rank correlation coefficient were used to evaluate the correlation of samples.

R Package stats (4.0.2) was used to evaluate the degree of gene variation, and the top 25% of genes were retained in terms of gene variation degree. The clustering algorithm of *k*‐means was further employed to divide these genes into multiple modules according to their expression patterns. Highly expressed genes were identified, and GO enrichment analysis was further performed by enrichGO and gseGO functions of clusterProfiler (3.16.1).^[^ ^106^ ^]^

The microarray data was processed by the R package GEOquery (2.64.2),^[^ ^107^ ^]^ and the above method was used to screen the top 25% genes for *k*‐means clustering, and selected cluster genes were analyzed by enrichment in the same way as described above.

### scRNA‐Seq Analysis for Ovary and Testis

Upstream analysis of single‐cell data was performed by the standard procedures of CellRanger including alignment reads and digital expression matrices generation. The expression profiles of genes in each cell type were demonstrated by R package Seurat (4.1.1)^[^ ^108^ ^]^ through the process of clustering, dimension reduction (UMAP), differential analysis, and cell type recognition.

### Small RNA‐Seq Analysis for Detecting piRNA

For small RNA‐seq, three gonads of wildtype or five gonads of *pld6* ^−/−^ were mixed as one sample. A total of three biological replicates were performed. For each sample, 200 ng total RNA from the gonads of 22 dpf wildtype or *pld6^−/−^* was used for synthesis and amplification of cDNA, and 1 ng of amplified cDNA was used for construction of sequencing library using VAHTS TM Small RNA Library Prep Kit for Illumina (Vazyme NR801). VAHTS DNA Clean Beads and gel electrophoresis (6% polyacrylamide gel) were used for size selection of small RNA library. For analysis, quality control, and adapter removal were performed by Fastp (0.12.4) to obtain clean data, and seqkit (2.0.0) was used to remove repeated sequences and intercept specific‐length sequences. Sequence reads were mapped to miRNA and piRNA sequences by Bowtie2 (2.2.5). Finally, the percentages of the first and tenth bases in the wildtype and mutants were calculated to obtain base preference information.

### Statistical Analysis

Significance of differences between means was analyzed using two‐sided *t*‐test. Sample sizes were indicated in the figures or figure legends. Plotted mean was calculated by GraphPad software. Data were shown as mean ± SD. *P* value below 0.05 marked as *, *P* value below 0.01 marked as **, and *P* value below 0.001 marked as ***; ns means no significant difference.

## Conflict of Interest

The authors declare no conflict of interest.

## Supporting information

Supporting Information

## Data Availability

The data that support the findings of this study are available in the supplementary material of this article.
