# Supplementary material for: A Germline‐Specific Regulator of Mitochondrial Fusion is Required for Maintenance and Differentiation of Germline Stem and Progenitor Cells
Source: Adv Sci (Weinh). 2022 Oct 18;9(36):2203631. doi: 10.1002/advs.202203631 (PMC9798980; doi:10.1002/advs.202203631)
Supplement: Supplementary file 1 — Supporting Information [file ADVS-9-2203631-s001.pdf]

ADVANCED SCIENCE
Open Access

Supporting Information

for *Adv. Sci.*, DOI 10.1002/advs.202203631

A Germline-Specific Regulator of Mitochondrial Fusion is Required for Maintenance and Differentiation of Germline Stem and Progenitor Cells

Ru Zhang, Yi-Xuan Tu, Ding Ye, Zhenglong Gu, Zhen-Xia Chen and Yonghua Sun**

Supplemental Materials

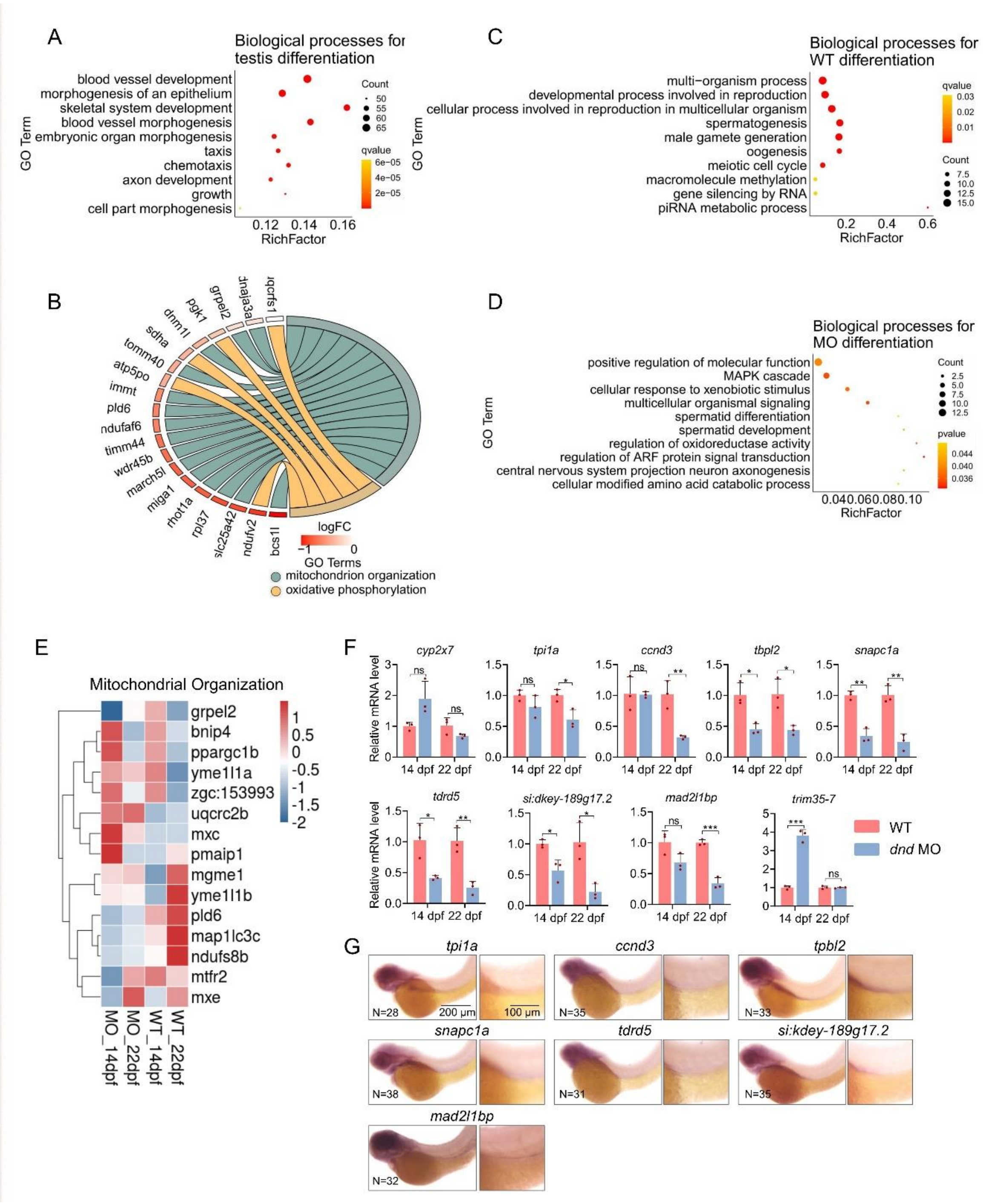

Figure S1

(A) GO enrichment analysis of cluster 1 (in Figure 1A)

(B) Expression of the main differential genes related to mitochondrion organization and oxidative phosphorylation

(C) GO enrichment analysis of cluster 4 (in Figure 1E)

(D) GO enrichment analysis of cluster 3 (in Figure 1E)

(E) Heatmap of mitochondrion organization-related genes in wildtype and *dnd* MO

(F) RT-qPCR verification of the differential gene expression in gonads of wildtype and *dnd* MO. Every three gonads were mixed into a sample and three biological replicates were performed.

(G) Detection of PGC-specific gene expression by WISH on 3 dpf embryos. N represents analyzed embryo number.

The data were expressed as mean ± SD. The *P* values in this figure were calculated by two-sided *t*-test. *, $P < 0.05$; **, $P < 0.01$; ***, $P < 0.001$; ns, no significant difference; MO, morpholino; WISH, whole-mount *in situ* hybridization; dpf, days post fertilization; RT-qPCR, reverse-transcription quantitative PCR.

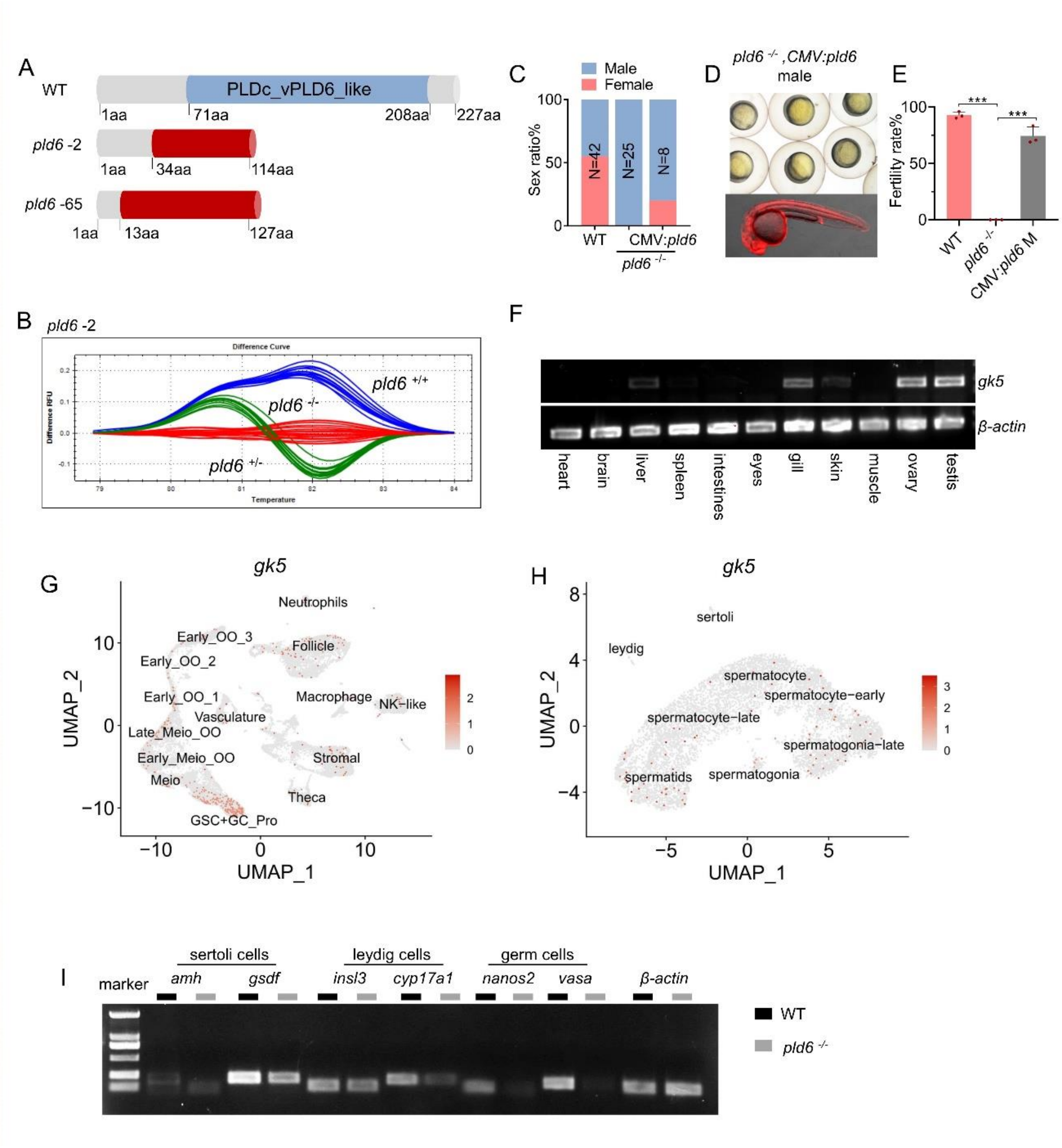

Figure S2

(A) Schematic of truncated protein in *pld6* mutant

(B) Identification of wildtype, heterozygous mutant, and homozygous mutant (2 bp deletion) of *pld6* by HRM

(C) Sex ratio of wildtype, *pld6*$^{-/-}$, CMV: *pld6* transgenic *pld6*$^{-/-}$ groups. N represents analyzed embryo number.

(D) Morphological observation of embryos obtained from CMV: *pld6* transgenic *pld6*$^{-/-}$ male mated with wildtype female

(E) Fertilization rates of wildtype, *pld6* $^{-/-}$, CMV: *pld6* transgenic *pld6*$^{-/-}$ males

(F) Expression detection of *gk5* in different tissues by qPCR.

(G) The distribution of *gk5*-expressed cells in ovary

(H) The distribution of *gk5*-expressed cells in testis

(I) RT-PCR detection of molecular markers of different cell types in wildtype and mutant testes

The data were expressed as mean ± SD. The *P* values in this figure were calculated by two-sided *t*-test. ***, $P < 0.001$; HRM, high resolution melting analysis; RT-PCR, reverse-transcription PCR.

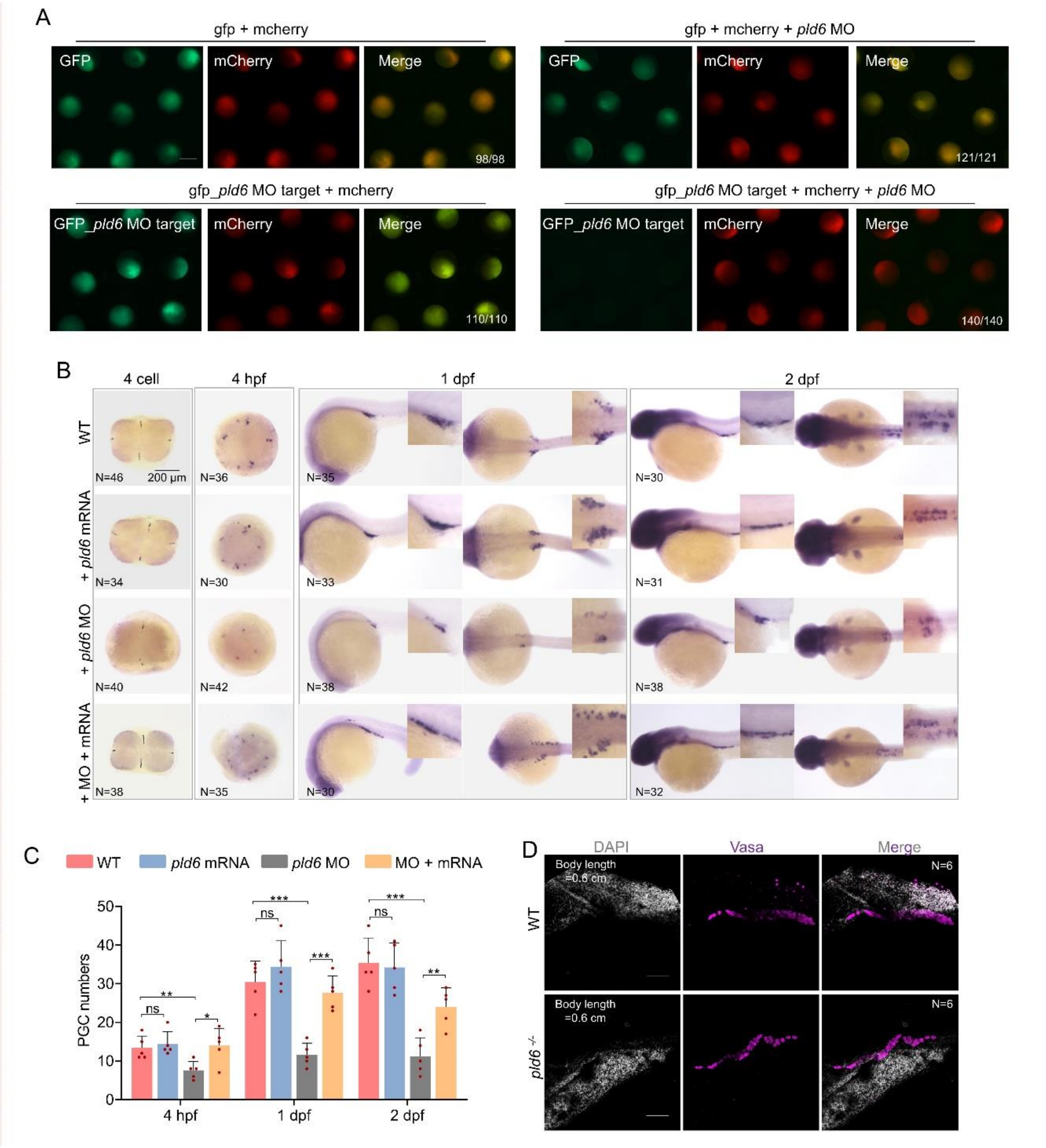

Figure S3

(A) Efficiency test of morpholino targeting zebrafish *pld6* ATG by fluorescence intensity. GFP was used as negative control. mCherry was used to normalize. The numbers below the pictures mean number of embryos showing representative fluorescence intensity/total number of embryos. Scale bar: 500 μm.

(B) Comparison of PGC numbers between wildtype, *pld6*-overexpressing, *pld6*-knockdown and *pld6* mRNA co-injected *pld6*-knockdown embryos at 4-cell, 4 hpf, 1 dpf, and 2 dpf stages by WISH. N represents analyzed embryo number.

(C) Statistical analysis of total PGC numbers in embryos in panel B

(D) Tracing of germ cell development in wildtype and *pld6*$^{-/-}$ juvenile gonads at 14 dpf. Anti-Vasa staining was performed to label the germ cells, and DAPI staining was performed to label the nuclear. Scale bar: 100 μm.

The data were expressed as mean ± SD. The *P* values in this figure were calculated by two-sided *t*-test. *, $P < 0.05$; **, $P < 0.01$; ***, $P < 0.001$; ns, no significant difference; WISH, whole-mount *in situ* hybridization; hpf, hours post fertilization; dpf, days post fertilization.

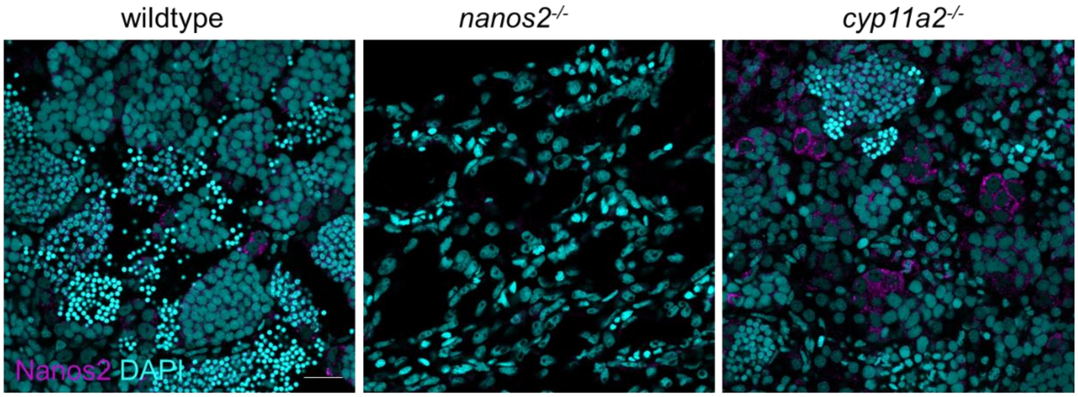

Figure S4

Immunofluorescence staining with Nanos2 antibody of the testis of *nanos2*$^{-/-}$, *cyp11a2*$^{-/-}$ at 4 mpf. N represents analyzed individual number. Scale bar：20 μm. mpf, months post fertilization.

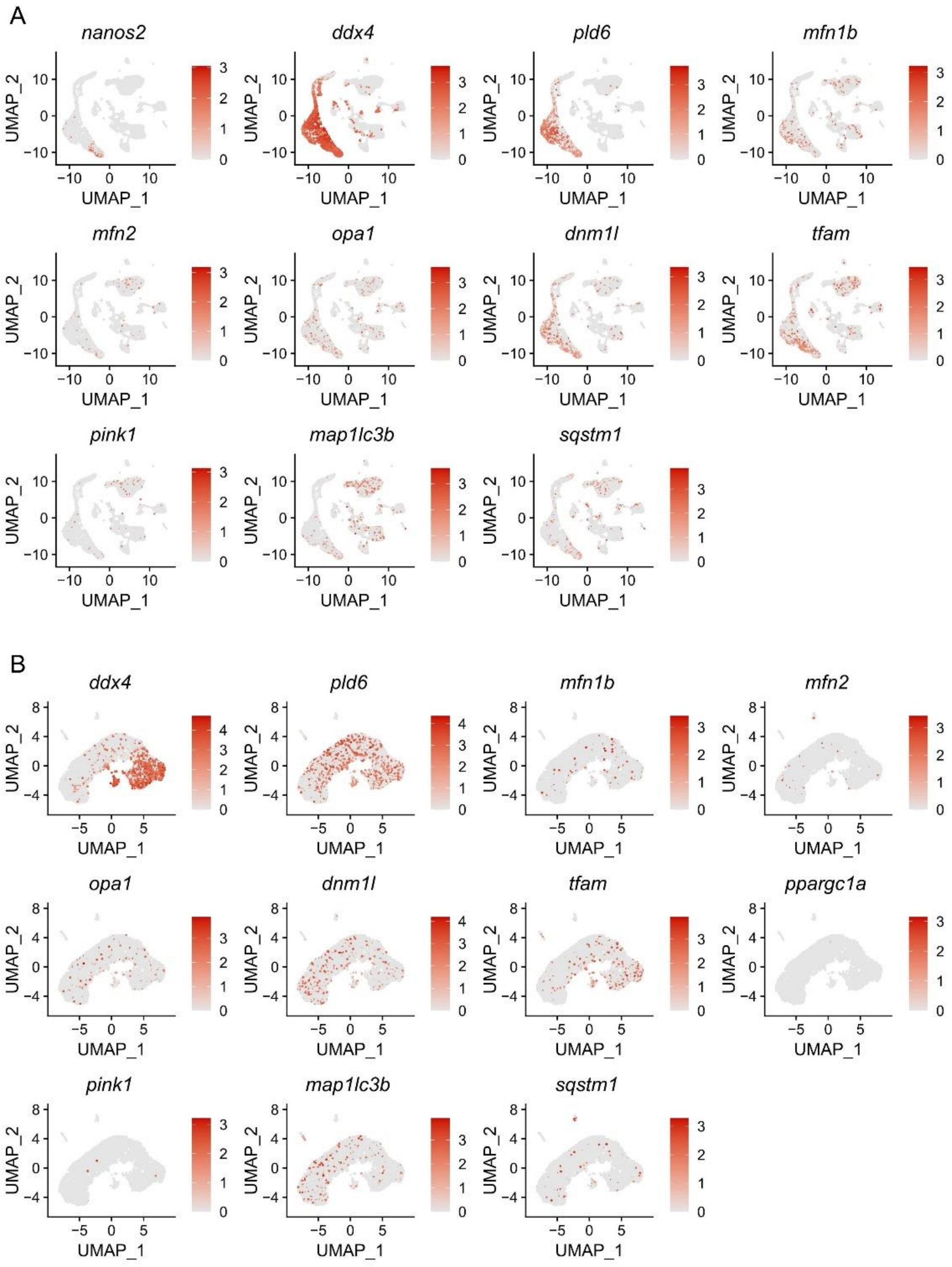

Figure S5

(A) The expression features of mitochondrial physiology related genes (*pld6*, *mfn1b*, *mfn2*, *opa1*, *dnm1l*, *tfam*, *pink1*, *map1lc3b* and *sqstm1*) across the different cell types in ovary. *nanos2* was used to indicate germline stem cells. *ddx4* was used to indicate germ cells.

(B) The expression features of mitochondrial physiology related genes (*pld6*, *mfn1b*, *mfn2*, *opa1*, *dnm1l*, *tfam*, *ppargc1a*, *pink1*, *map1lc3b* and *sqstm1*) across the different cell types in testis. *ddx4* was used to indicate germ cells.

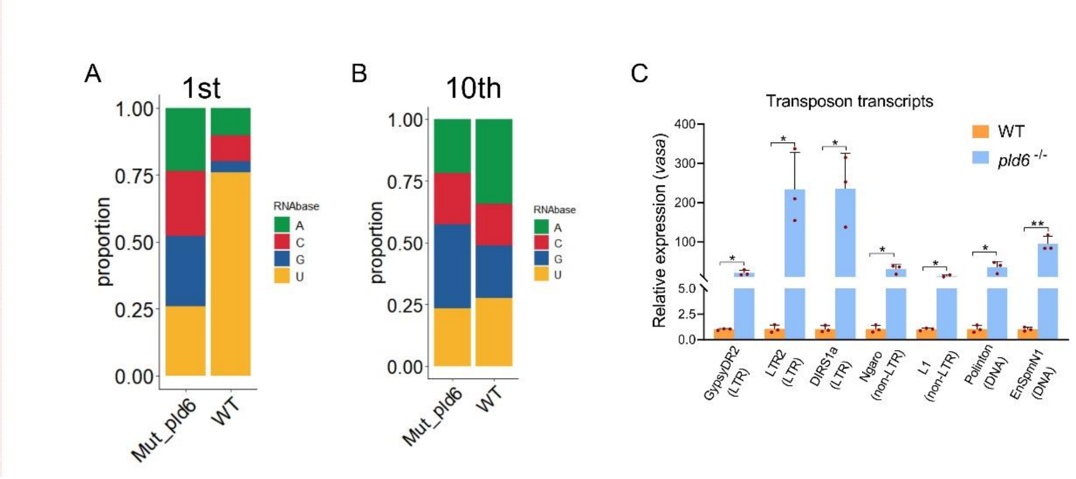

Figure S6

(A) Nucleotide composition of the 1st bases of piRNA from wildtype and *pld6*$^{-/-}$ mutant gonads

(B) Nucleotide composition of the 10th bases of piRNA from wildtype and *pld6*$^{-/-}$ mutant gonads

(C) Expression of transposons (*GypsyDR2*, *LTR*, *DIRS1a*, *Ngaro*, *L1*, *Polinton* and *EnSpmN1*) in wildtype and *pld6*$^{-/-}$ juvenile gonads. Germ cell marker *vasa* was used as a reference gene to avoid the influence of germ cell number difference in wildtype and *pld6*$^{-/-}$.

The data were expressed as mean ± SD. The *P* values in this figure were calculated by two-sided *t*-test. *, $P < 0.05$; **, $P < 0.01$.

S1 Table

The primers for mutant screening.

gene	Primer name	sequence（5’−3’）
z-*pld6*	Cas9-F	TGATTTGTGACTGACTGTTTAC
	Cas9-R	CGCTTGTGTGACTTTGGTA
	Melt test-F	TGTTGCGTTTGTTCTGGG
	Melt test-R	GACGCAGACTTGAGGTGAA
z-*tp53*	Cas9-F	TTACATGAAATTGCCAGAGTATG
	Cas9-R	CAACTGAACTGTTTCGGATAGC

S2 Table

The primers for RT-qPCR.

primers	Sequence
pld6-RT-F	5’- TGGGTGTGGAGTGGTTAAA-3’
pld6-RT-R	5’-TGCCGTGAGGAAGAGGAC -3’
β-actin-RT-F	5’-GACATCAAGGAGAAGCTGTGC-3’
β-actin -RT-R	5’-GAGGAGGGCAAAGTGGTAAAC-3’
cyp2x7-RT-F	5’-CCTTCATACCCTTCTCCAC-3’
cyp2x7-RT-R	5’-ATGGTGAGCCCAAATACA-3’
mgmt-RT-F	5’-GACTGGCTTCAATGCTACTT-3’
mgmt-RT-R	5’-GTTTTACCAACTCCCACTTC-3’
pex10-RT-F	5’-ATCAGGTCAGTCAGTCCTCGTC-3’
pex10-RT-R	5’-CGGGCATTCATTCTTGGTAT-3’
tpi1a-RT-F	5’-ATGAACGGCGATAAAGAGT-3’
tpi1a-RT-R	5’-CTCCTTTGGGCACCTTAT-3’
ccnd3-RT-F	5’-GGAGAAGCCGAACATCAG-3’
ccnd3-RT-R	5’-CACGGCACTCTGGGACAT-3’
brf2-RT-F	5’-GTGCGGCTCATCTCGTGT-3’
brf2-RT-R	5’-GTAGAGGTGAAAAACGGCA-3’
ctc1-RT-F	5’-CTTGACGGACAGGTGGG-3’
ctc1-RT-R	5’-ACGCCGATGCCTGATAC-3’
tbpl2-RT-F	5’-CGCAGAAAGAGTCCACACA-3’
tbpl2-RT-R	5’-GGGCAAGCAAGATTCACAG-3’
snapc1a-RT-F	5’-TTTTCGGCTATCTGGAGG-3’
snapc1a-RT-R	5’-AGAAGGTAACTGTAGGCTGTG-3’
tdrd5-RT-F	5’-GCACCTCGCCCTCATACA-3’
tdrd5-RT-R	5’-GATGCCGTCCGACAAGAA-3’
zgc:103482-RT-F	5’-GAGCCAAAGAAAACTTCAACC-3’
zgc:103482-RT-R	5’-TTCAACAGAGAAACTGAGGGG-3’
org-RT-F	5’-CAGTCTGTACCAGCATCGG-3’
org-RT-R	5’-ACCTCCTCGTCCTCATCC-3’
sept4a-RT-F	5’-TGTGCCTCTGGTGCCTGT-3’

sept4a-RT-R	5'-TGTCTTTGACGCATCTGTTCT-3'
trim35-7-RT-F	5'-TCTTTGTTTCCAGACCCAC-3'
trim35-7-RT-R	5'-CAGGAGTGTAGTGGACGATGT-3'
chtopb-RT-F	5'-ACCGCTTCACCAGTTTGC-3'
chtopb-RT-R	5'-CTCGCCTTCACGTTCATG-3'
si:dkey-189g17.2-RT-F	5'-TGTTGATGAGGTCACTGTTTG-3'
si:dkey-189g17.2-RT-R	5'-AAGCCCTCCTTCGTTTTGTA-3'
mad2l1bp-RT-F	5'-GCAGGACGACATTGGAGA-3'
mad2l1bp-RT-R	5'-ATCTACAGAGTGTGGAGGCA-3'
frmd3-RT-F	5'-AACAGAGGCATTGGTTGGAC-3'
frmd3-RT-R	5'-TGGGCTCCTGAGGGTAGA-3'
ildr1b-RT-F	5'-GGAACGGAGTACCGACAG-3'
ildr1b-RT-R	5'-TGAGCAGCACAGTTAGCC-3'
rnf17-RT-F	5'-CAGCAGCCAGGAGGATTA-3'
rnf17-RT-R	5'-CATTTGGCGTGCGGATAT-3'
gk5-RT-F	5'-GGGATGGCTTCGTCACTG-3'
gk5-RT-R	5'-CCGTTCCAGGACCTCACC-3'
dnml1-RT-F	5'-AGCCAGTCAGGTGATCGCCGA-3'
dnml1-RT- R	5'-CGCAGGGTTCGCGTGAAGGG-3'
mfn1b-RT- F	5'-CTGGGTCCCGTCAACGCCAA-3'
mfn1b-RT-R	5'-ACTGAACCACCGCTGGGGCT-3'
opa1-RT- F	5'-GCCGGAAGTGTAGTTACCTG-3'
opa1-RT- R	5'-AGGTGGTCTCTGTGGGTTGT-3'
ppargc1a-RT-F	5'-GGCCCAGCGAGCCAAACCAA-3'
ppargc1a -RT- R	5'-TGGCTTTGTGAGGAGGCGTGG-3'
mfn2-RT- F	5'-CTCTCGACTGCAAGAGCAAAATC-3'
mfn2-RT- R	5'-CACGACTGGTGAAGGATGGA-3'
pink1-RT- F	5'-TGGTGGGTGAACAGAGGG-3'
pink1-RT- R	5'-GTTTTTCCTGGTAACTCCGA-3'
sqstm1-RT- F	5'-CTGAAAACCCAAATGAGAAT-3'
sqstm1-RT- R	5'-GGGGTTACTTTGGTCCGC-3'
map1lc2b-RT- F	5'-ACATTTGAGCAGCGGGTG-3'
map1lc2b -RT- R	5'-TGGTTGGAGTTGAGTTGG-3'
GypsyDR2-RT-F	5'-GAAATCACCTGTGCATTTAC-3'
GypsyDR2-RT-R	5'-ATGCAGACATTGGGTAAAGC-3'
EnSpmN1-RT-F	5'-GATTGGCCATTGTGTTCACATGC
EnSpmN-RT-R	5'-GCTGTGACTGTCATAGGTTTACC-3'
Ngaro-RT-F	5'-GGAGCGATCGAGACCTACC-3'
Ngaro-RT-R	5'-CAATCATATCACGTGCTCCTCTCG-3'
Polinton-RT-F	5'-CCTGACAATGTTGTCAGCCTG-3'
Polinton-RT-R	5'-CATGAAAGCTAAGGGTATAACTCTG-3'
DIRS1a-RT-F	5'-GGGTGCGTCACGCTTGC-3'
DIRS1a-RT-R	5'-GTAACCTCGAACGTTCCCC-3'
L1-5-RT-F	5'-GCACAAAGGACAAATTCACTGGAC-3'
L1-5-RT-R	5'-GTCCACGTTTAGTATTACAGTTGC-3'
LTR2-RT-F	5'-GGTGTCGTTAGAATGCCCTTGAC-3'
LTR2-RT-R	5'-GGTTATACCTGTGGGTCACGTG-3'
